# TT-PADM: A Time-Driven Transformer Diffusion Model for Robust Sparse-View and Limited-View Photoacoustic Tomography

**DOI:** 10.34133/bmef.0237

**Published:** 2026-03-02

**Authors:** Jiawei Zheng, Wende Dong, Junjun Sun, Qingfei Song, Xiaohua Jiang, Sheng Wang, Songde Liu, Chao Tian

**Affiliations:** ^1^ Department of Anesthesiology, the First Affiliated Hospital of USTC, Division of Life Sciences and Medicine, University of Science and Technology of China, Hefei, Anhui 230001, China.; ^2^ Institute of Advanced Technology, University of Science and Technology of China, Hefei, Anhui 230026, China.; ^3^ Institute of Artificial Intelligence, Hefei Comprehensive National Science Center, Hefei, Anhui 230088, China.; ^4^ College of Automation Engineering, Nanjing University of Aeronautics and Astronautics, Nanjing, Jiangsu 211106, China.; ^5^ School of Artificial Intelligence, Anhui University, Hefei, Anhui 230601, China.; ^6^ School of Engineering Science, University of Science and Technology of China, Hefei, Anhui 230026, China.; ^7^ Department of Obstetrics and Gynecology, the First Affiliated Hospital of USTC, Division of Life Sciences and Medicine, University of Science and Technology of China, Hefei, Anhui, 230001, China.; ^8^ Anhui Province Key Laboratory of Biomedical Imaging and Intelligent Processing, Institute of Artificial Intelligence, Hefei Comprehensive National Science Center, Hefei, Anhui 230088, China.

## Abstract

**Objective:** To develop a high-performance reconstruction framework that enables high-quality photoacoustic tomography (PAT) imaging under limited-view and sparse-view acquisition constraints. **Impact Statement:** The proposed method reduces the number of required acoustic transducers while maintaining image quality comparable to full-view systems, providing a practical and cost-efficient solution for biomedical PAT imaging. **Introduction:** PAT offers high-resolution visualization of biological tissues. However, restrictions such as reduced transducer counts or incomplete detection geometries render the inverse problem severely ill-posed, leading to marked degradation in reconstructed images. Although diffusion models have recently shown strong promise for image restoration, existing architectures can be computationally intensive or insufficiently expressive for the complexities of PAT.**Methods:** We introduce a time-driven transformer-based photoacoustic diffusion model (TT-PADM) that directly restores high-quality images from limited-view and sparse-view PAT reconstructions. TT-PADM uses a time-driven transformer within a time-dependent noise-estimation network, reducing model parameters by over 80% relative to conventional transformer designs while enhancing the generative capacity of the diffusion process. **Results:** Simulations and experimental results show that TT-PADM delivers high-fidelity reconstructions even under severely limited acquisition conditions, producing image quality comparable to full-view PAT systems. Quantitative and qualitative analyses show that TT-PADM consistently surpasses state-of-the-art reconstruction approaches, providing notable improvements in structural accuracy and noise suppression. **Conclusion:** TT-PADM offers a robust, parameter-efficient, and highly effective solution for PAT image restoration under practical hardware constraints, with strong potential for deployment in resource-limited biomedical imaging scenarios.

## Introduction

Photoacoustic tomography (PAT) is an innovative biomedical imaging modality that combines the advantages of rich optical contrast and high acoustic resolution, enabling noninvasive, cost-effective imaging of biological tissues [1–3]. Under nanosecond laser pulse irradiation, biological tissues absorb laser energy, resulting in a rapid temperature increase that induces the emission of ultrasound signals via the thermoelastic effect. These photoacoustic signals are subsequently detected by a surrounding array of ultrasound transducers, which are used to reconstruct an image representing the distribution of optical absorption. Over the past 2 decades, PAT has demonstrated substantial potential in both preclinical animal models [4] and clinical applications [5–7], presenting promising prospects for broader adoption in biomedical imaging.

Image reconstruction is a critical step in PAT image formation. Conventional PAT image reconstruction algorithms can be broadly categorized into 5 types [8,9]: back projection [10], delay and sum [11], series expansion [12,13], time reversal [14,15], and iterative reconstruction (IR) [16,17]. In general, high-quality PAT images can be accurately reconstructed when the detection transducer array fully encloses the tissue and contains a sufficiently large number of elements. However, in practical applications, constructing an imaging system that meets these ideal specifications is often prohibitively expensive, and in many in vivo scenarios, the sensor array typically only partially surrounds the tissue. Previous studies have demonstrated that with sparse transducer arrays or incomplete arrays, PAT images are susceptible to artifacts and structural distortions [18–21]. Tian et al. conducted a comparative analysis of various reconstruction algorithms under sparse-view (reduced transducer elements) and limited-view (incomplete angular coverage of the detection array) imaging conditions [8]. Their results indicated that back projection, delay and sum, series expansion, and time reversal require a larger number of projections to achieve acceptable image quality. In contrast, IR methods, which combine a least-squares model with regularization techniques such as total variation to mitigate artifacts, can yield improved image quality but are limited in reconstruction speed due to the need to compute the entire full photoacoustic field [22].

Recent advances in deep learning have demonstrated considerable potential for PAT image processing, particularly in addressing sparse-view and limited-view problems. Most prior studies have used a postprocessing approach, training deep learning networks—such as convolutional neural networks [23,24], U-Net [25,26], or generative adversarial networks (GANs) [27,28]—to directly reconstruct full-view images from their spatially aliased counterparts. These postprocessing networks typically operate in the image domain, rather than leveraging the richer information contained in raw photoacoustic data, which results in reduced computational complexity but at the cost of losing fine-grained structural details inherent in the signal domain. To enhance image quality, other researchers have proposed data-driven networks designed to directly solve the inverse problem [29] or have combined well-designed networks with model-based IR approaches [30]. While deep learning networks have demonstrated substantial performance improvements in PAT image enhancement, challenges remain in achieving realistic enhancement under nonideal conditions, due to limitations in network generalization capacity.

Recent advancements in diffusion models, including the score-based generative model (SGM) [31] and the denoising diffusion probabilistic model (DDPM) [32], have garnered considerable attention for image generation and restoration tasks. Diffusion models offer distinct advantages in image processing by leveraging gradient fields of the data distribution to iteratively refine images, enabling high-quality detail restoration and robust noise handling through a unified probabilistic framework. These models have been successfully applied in biomedical imaging, demonstrating their effectiveness across a range of applications. Among generative models, the SGM, which is based on stochastic differential equations (SDEs), implements a more efficient sampling approach, thereby enhancing generative capacity. The first application of SGM to biomedical imaging involved generating magnetic resonance imaging (MRI) images using a Langevin solver [33]. Subsequently, several studies have explored the use of diffusion models for computed tomography [34,35], MRI [32,36], and PAT [37,38] image reconstructions, achieving state-of-the-art results in image quality.

In this work, we propose a time-driven transformer-based photoacoustic diffusion model (TT-PADM) for high-quality image enhancement in sparse-view and limited-view PAT. Building upon the SGM framework, TT-PADM progressively restores high-quality images from spatially undersampled photoacoustic signals via SDEs. The model integrates a time-driven transformer architecture that incorporates the diffusion time step at each stage of the reverse process, enabling temporally coherent feature refinement. In addition, TT-PADM includes a lightweight time-driven multihead transposed attention (TMTA) mechanism and a gated convolutional feedforward network (GCFN), which efficiently capture global context while maintaining computational efficiency. Extensive evaluations on both simulated and in vivo data demonstrate that TT-PADM outperforms existing state-of-the-art deep-learning-based methods in addressing sparse- and limited-view problems.

The structure of this paper is organized as follows. Results presents the results of simulation and in vivo experiments. Discussion and Conclusion provides the conclusion. Materials and Methods presents an overview of the mathematical foundations underlying both the acoustic forward propagation and image reconstruction problems in PAT and introduces the TT-PADM.

## Results

In this section, we present the results obtained from our proposed model on both simulation and in vivo datasets. We assess the performance of the TT-PADM model on 2 transducer configurations: sparse-view and limited-view arrays. Notably, restoring limited-view images presents a more substantial challenge due to the reduced angular coverage. To evaluate the performance of the proposed TT-PADM method, we conduct comparative analyses against 2 representative deep learning approaches, i.e., FD-UNet [39] and LV-GAN [40]. FD-UNet, a widely adopted baseline, is selected as a representative U-Net-based architecture commonly used in PAT image enhancement. LV-GAN, a specialized GAN for limited-view PAT enhancement, is also considered. The quantitative performance of these methods is assessed using 3 widely recognized metrics: peak signal-to-noise ratio (PSNR), root mean square error (RMSE), and structural similarity index measure (SSIM) (see Note S2).

### TT-PADM achieves high-quality image restoration in mouse embryo simulation

To rigorously evaluate the performance of the proposed model, we constructed a numerical dataset comprising optical projection tomography images of mouse embryo slices [41]. This dataset includes a total of 2,878 images derived from 51 distinct mouse embryos. The images were randomly partitioned into 3 subsets: 80% for training, 10% for validation, and 10% for testing.

**Fig. 1.**
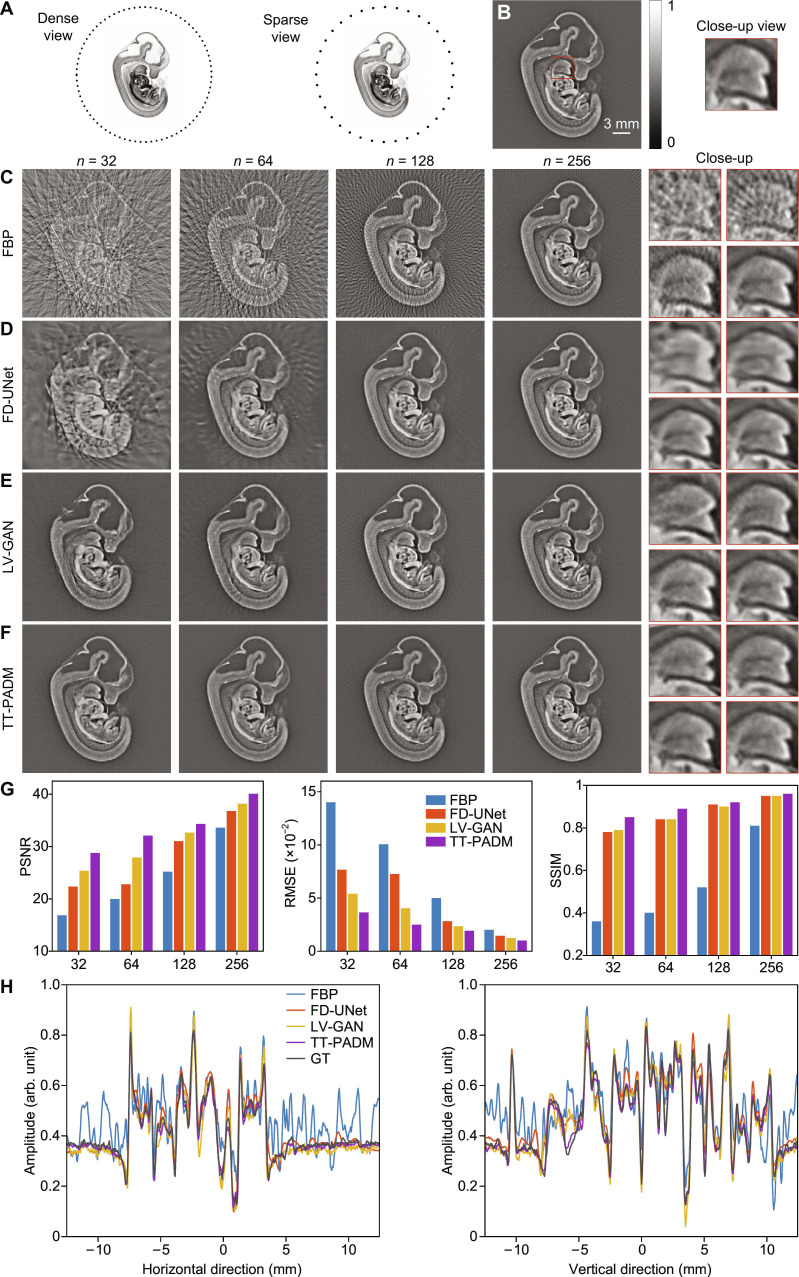
Restoration of mouse embryo images under sparse-view imaging conditions. (A) Schematic diagram showing the configuration of the mouse embryo imaging simulation under dense-view and sparse-view conditions. (B) Ground-truth image, with a region of interest (ROI) marked by a red box. The panel on the right displays a magnified view of the ROI. (C) Reconstructions using the FBP method with increasing transducer elements (*n* = 32, 64, 128, and 256). The 4 panels on the right show magnified details corresponding to each sparse-view case. (D to F) Restored images by FD-UNet, LV-GAN, and TT-PADM, respectively. For each method, the 4 panels on the right display magnified ROIs (top left, *n* = 32; top right, *n* = 64; bottom left, *n* = 128; bottom right, *n* = 256), corresponding to the same position as the red box in (B). (G) Quantitative evaluation of the results on the test dataset. (H) Comparison of intensity profiles along the horizontal and vertical lines at the center of the images for the 64-view case. GT, ground truth.

We first evaluated the performance of TT-PADM for image enhancement in sparse-view PAT. Figure 1A illustrates the geometry of the transducer array, which is circular in shape and contains 512 elements. Sparse-view photoacoustic signals generated from mouse embryos are detected by 256, 128, 64, and 32 transducers, respectively, with the 512-element images serving as the reference (Fig. 1B). Sparse-view images reconstructed using the filtered back-projection (FBP) algorithm (500 × 500 pixels) exhibit distortions, including streak artifacts (Fig. 1C). While existing deep learning methods, such as FD-UNet and LV-GAN, provide partial artifact suppression, their performance markedly deteriorates under highly sparse sampling conditions, particularly when only 32 or 64 transducer elements are used (Fig. 1D and E). In contrast, TT-PADM excels in both artifact removal and structural recovery across all sparse-view configurations (Fig. 1F) and shows performance improvements over the original SGM (see Note S3). The red boxes in Fig. 1C to F clearly show that TT-PADM outperforms the alternative methods in preserving fine details and restoring structural integrity across all sparse-view scenarios. Quantitative evaluations further substantiate its superiority, with TT-PADM achieving the highest PSNR, lowest RMSE, and highest SSIM scores (Fig. 1G). The close alignment between TT-PADM’s intensity profiles along the horizontal and vertical lines in the middle of the images and the ground truth in the 64-view case (Fig. 1H) further validates its accurate pixel-level reconstruction capability.

**Fig. 2.**
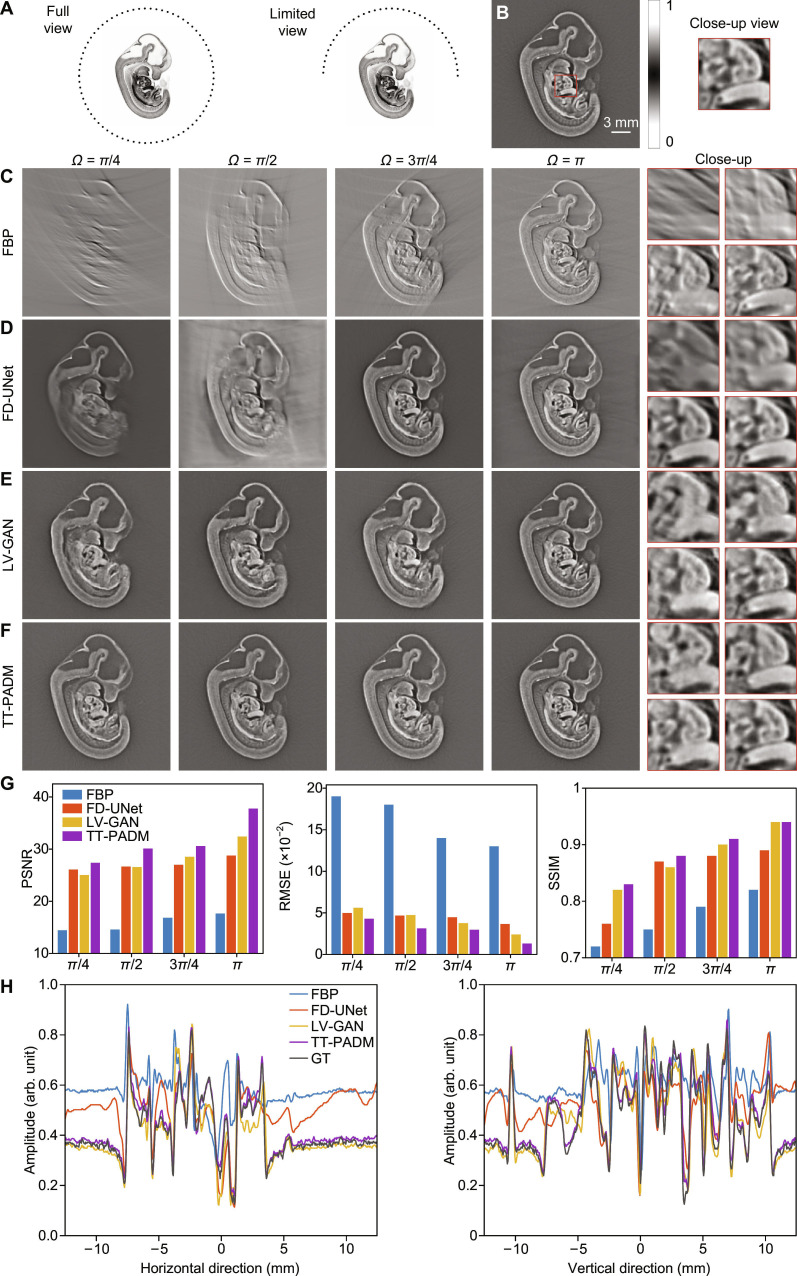
Restoration of mouse embryo images under limited-view imaging conditions. (A) Schematic diagram illustrating the configuration of the mouse embryo imaging simulation under full-view and limited-view conditions. (B) Ground-truth image, with an ROI highlighted by a red box. The panel on the right shows a magnified view of this ROI. (C) Reconstructions using the FBP method with reduced angular coverage (*Ω* = *π*/4, *π*/2, 3*π*/4, and *π*). The 4 panels on the right show magnified details corresponding to each view angle. (D to F) Restored images by FD-UNet, LV-GAN, and TT-PADM, respectively. For each method, the 4 panels on the right display magnified ROIs (top left, *Ω* = *π*/4; top right, *Ω* = *π*/2; bottom left, *Ω* = 3*π*/4; bottom right, *Ω* = *π*), corresponding to the same region marked by the red box in (B). (G) Quantitative evaluation of the results on the test dataset. (H) Comparison of intensity profiles along the horizontal and vertical axes at the center of the images for the *π*/2-view case.

For limited-view reconstruction, the angular coverage of the circular detection array was progressively reduced from a full circle (2*π*) to *π*, 3*π*/4, *π*/2, and *π*/4, as shown in Fig. 2A. Images reconstructed from 512-element full-view signals using FBP (500 × 500 pixels) were used as the ground truth (Fig. 2B). Compared to sparse-view scenarios, limited-view FBP reconstructions suffer from more severe structural distortion due to substantial angular information loss (Fig. 2C), posing a fundamentally more challenging reconstruction problem. Among the existing approaches, FD-UNet fails to recover boundary details when the view angle falls below 3*π*/4 (Fig. 2D), while LV-GAN, although performing marginally better, still produces blurred structural features in critical regions (see the region of interest marked by the red boxes in the right side of Fig. 2E). In contrast, TT-PADM demonstrates remarkable capability in preserving fine details and mitigating structural loss, particularly under the extreme *π*/4 viewing condition (Fig. 2F), and shows performance improvements over the original SGM (see Note S3). Quantitative evaluations confirm this advantage, with TT-PADM achieving superior PSNR, RMSE, and SSIM values across all limited-view cases (Fig. 2G). Comparison of the intensity profiles along the horizontal and vertical axes at the center of the images in *π*/2-view case (Fig. 2H) reveals close alignment between TT-PADM-enhanced images and the ground truth, further validating its pixel-level accuracy.

### TT-PADM achieves high-quality image restoration in in vivo mouse imaging

To substantiate the efficacy of TT-PADM, we extended its validation to the restoration of sparse-view and limited-view PAT images obtained from in vivo mouse experiments (see Fig. S1). The in vivo mouse dataset consists of PAT images acquired from a cohort of twelve mice, with 780 images obtained for each subject. Among these, 8 mice were allocated for training (2/3 of the image dataset), 2 mice were designated for validation (1/6 of the dataset), and the remaining 2 were reserved for testing (1/6 of the dataset).

**Fig. 3.**
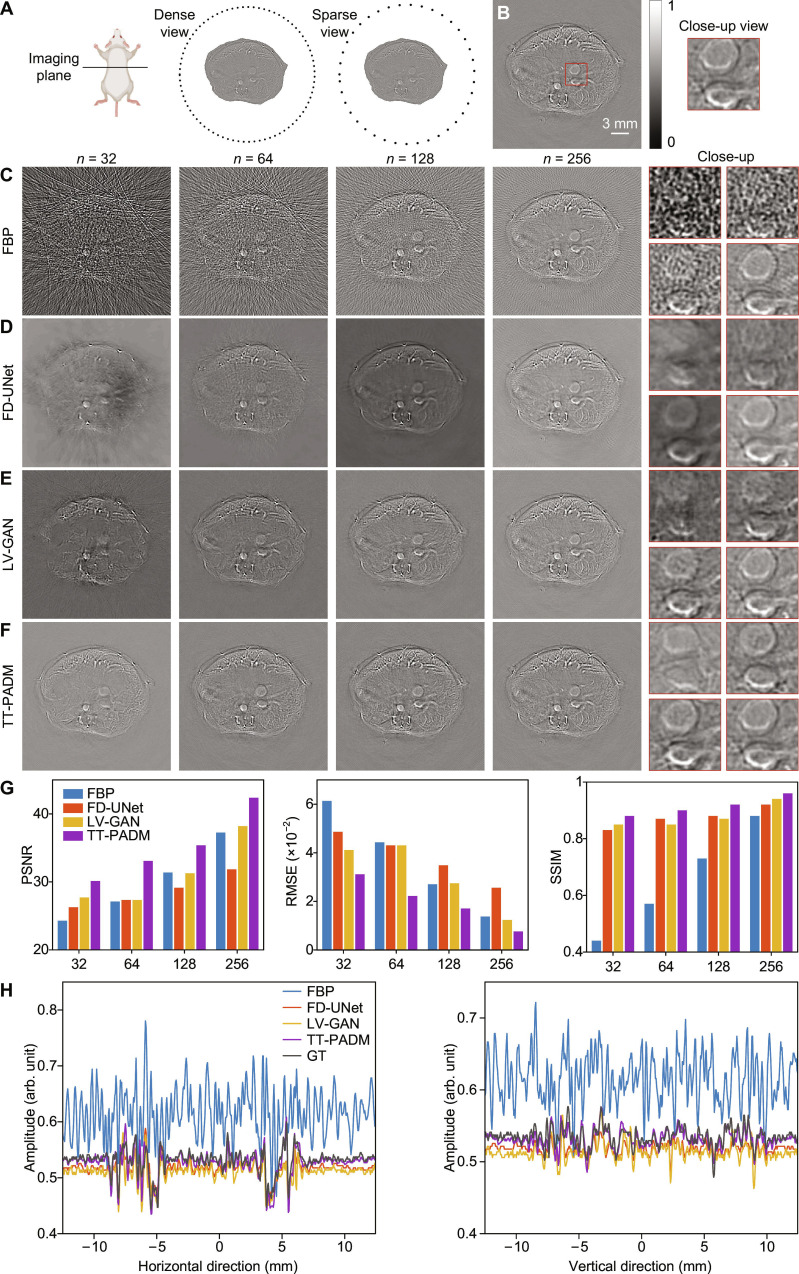
Restoration of in vivo mouse images under sparse-view imaging conditions. (A) Schematic diagram illustrating the configuration of the mouse experiment under dense-view and sparse-view conditions. (B) Ground-truth image, with an ROI highlighted by a red box. The panel on the right provides a magnified view of this ROI. (C) Reconstructions using the FBP method with reduced transducer elements (*n* = 32, 64, 128, and 256). The 4 panels on the right display magnified details corresponding to each sparse view case. (D to F) Images restored by FD-UNet, LV-GAN, and TT-PADM, respectively. For each method, the 4 panels on the right show magnified ROIs (top left, *n* = 32; top right, *n* = 64; bottom left, *n* = 128; bottom right, *n* = 256), corresponding to the same region marked by the red box in (B). (G) Quantitative evaluation of the results on the test dataset. (H) Comparison of intensity profiles along the horizontal and vertical axes at the center of the images for the 64-view case. Mouse image was created with BioRender.

For the sparse-view scenarios, a circular transducer array with 512 elements, identical to the one used in the previous simulation study, was used, as depicted in Fig. 3A. The number of transducer elements was progressively reduced from 512 to 256, 128, 64, and 32 elements, with 512-element images serving as the ground truth (Fig. 3B). Images reconstructed using FBP (500 × 500 pixels) suffer from pronounced streak artifacts and exhibit reduced quality (Fig. 3C). Except for the 256-element case, both FD-UNet and LV-GAN fail to adequately mitigate distortion within the mouse tissue, although external streak artifacts are moderately suppressed (Fig. 3D and E). In contrast, TT-PADM effectively suppresses streak artifacts and restores structural details, particularly in the 32- and 64-element cases (Fig. 3F). Furthermore, quantitative metrics, PSNR, RMSE, and SSIM, demonstrate that TT-PADM outperforms the other methods, achieving the highest and most robust performance on the in vivo mouse dataset (Fig. 3G). TT-PADM also shows performance improvements over the original SGM (see Note S4). Intensity profiles along the central horizontal and vertical axes of the images (Fig. 3H) show close agreement between TT-PADM reconstructions and the ground truth in the 64-view case. This result further validates the method’s reconstruction accuracy in practical imaging conditions.

**Fig. 4.**
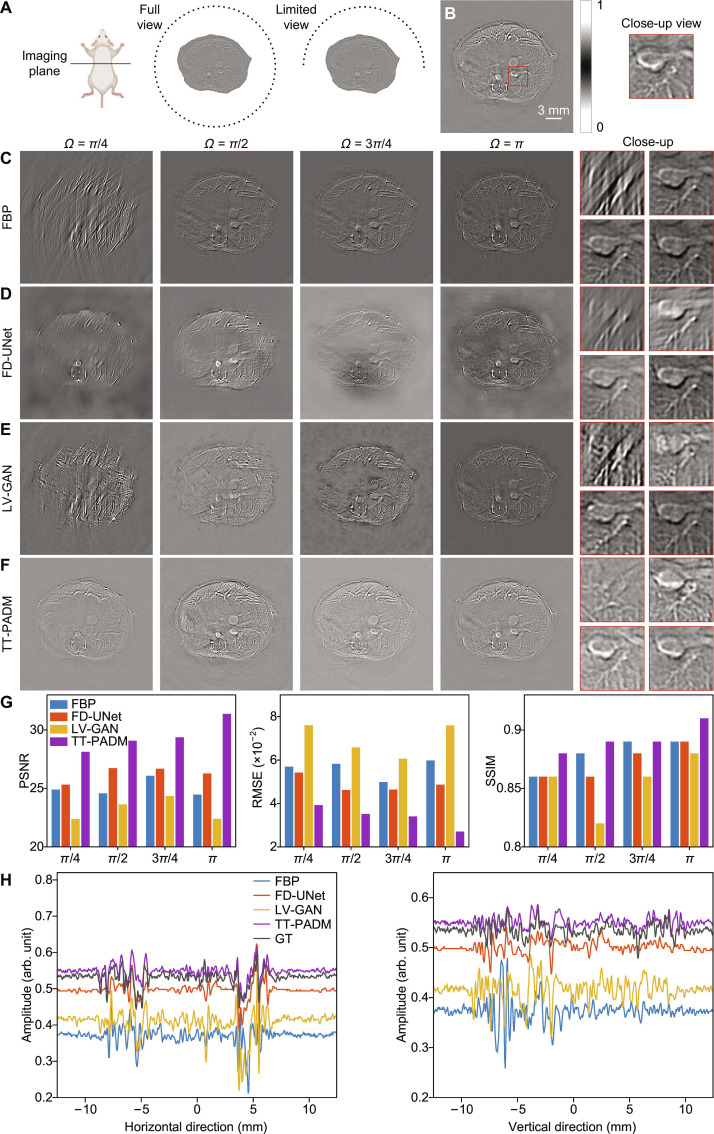
Restoration of in vivo mouse images under limited-view imaging conditions. (A) Schematic diagram illustrating the configuration of the mouse imaging experiment under full-view and limited-view conditions. (B) Ground-truth image, with an ROI marked by a red box. The panel on the right provides a magnified view of this ROI. (C) Reconstructions using the FBP method with reduced angular coverage (*Ω* = *π*/4, *π*/2, 3*π*/4, and *π*). The 4 panels on the right display magnified details corresponding to each view angle. (D to F) Enhanced results from FD-UNet, LV-GAN, and TT-PADM, respectively. For each method, the 4 panels on the right show magnified ROIs (top left, *Ω* = *π*/4; top right, *Ω* = *π*/2; bottom left, *Ω* = 3*π*/4; bottom right, *Ω* = *π*), corresponding to the same region marked by the red box in (B). (G) Quantitative evaluation of the results on the test dataset. (H) Comparison of intensity profiles along the horizontal and vertical axes at the center of the images for the *π*/2-view case.

For the limited-view experimental studies, the transducer array geometry (Fig. 4A) follows the simulation configuration, with angular coverage progressively reduced from full-view (2*π*) to *π*, 3*π*/4, *π*/2, and *π*/4. Corresponding full-view 512-element reconstructions serve as the ground truth (Fig. 4B). As the view angle decreases, images reconstructed via FBP (500 × 500 pixels) exhibit severe loss of structural information (Fig. 4C). The images restored by the other 3 models are presented in Fig. 4D to F. Results indicate that TT-PADM outperforms the other models in both background and feature restoration. In contrast, the images reconstructed by FD-UNet exhibit notable blurring and ink-like distortions resulting from the fixed local receptive fields of its convolutional layers, which fail to capture global pixel correlations [39], as well as from its limited feature extraction capacity that is susceptible to local optima [22], while LV-GAN struggles and fails to recover boundary details, particularly as the view angle decreases. In addition, as shown in the vessel regions in Fig. 4D to F, both FD-UNet and LV-GAN fail to recover vessel details under all limited-view scenarios. In contrast, TT-PADM demonstrates marked improvement in restoring vessel and boundary details, especially when the view angle is reduced to less than *π*/2. It also shows performance gains over the original SGM (see Note S4). As before, TT-PADM consistently achieves superior performance in terms of PSNR, RMSE, and SSIM (Fig. 4G). To further assess the restoration accuracy of different methods, Fig. 4H presents intensity profiles along the horizontal and vertical axes in the middle of the images for the *π*/2-view case. The intensity curve produced by TT-PADM closely matches the ground truth, further demonstrating its high restoration accuracy at the *π*/2 view angle.

### TT-PADM achieves high-quality image restoration in human finger imaging

To further demonstrate the efficacy of the proposed model, we conducted a study on a human experimental finger dataset. The experiment involved 5 volunteers and specifically focused on PAT images acquired from the index, middle, and ring fingers of each participant (see Fig. S1). A total of 180 to 200 PAT images were obtained from each of the aforementioned fingers, ensuring a comprehensive dataset for subsequent analysis. After initial data acquisition, a subset of images from 26 fingers was designated as the training set. Images from 2 distinct fingers were allocated for validation, and a separate set from 2 additional fingers was reserved for testing. In human finger PAT imaging, the presence of phalanges, as strongly heterogeneous tissue, can cause acoustic reflection, refraction, and scattering at interfaces. These phenomena can lead to artifacts and distortion in reconstructed PAT images, especially in limited-view scenarios [42].

**Fig. 5.**
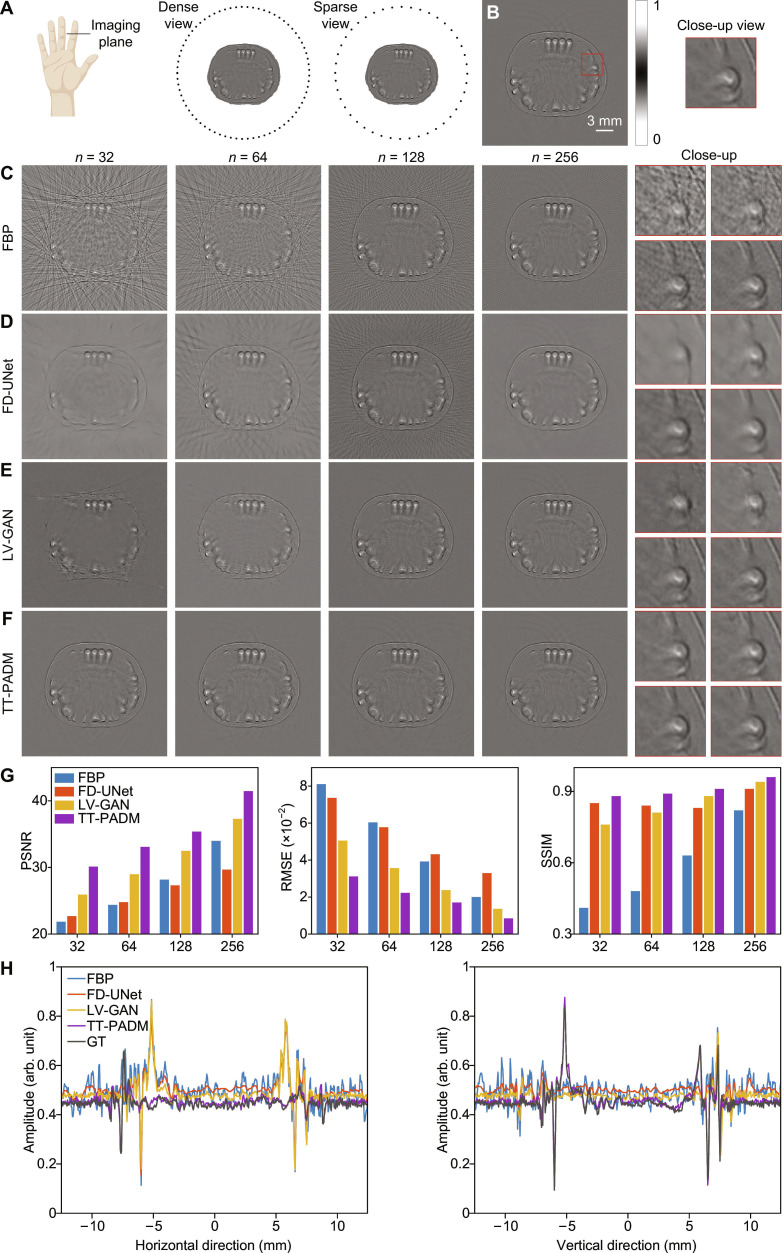
Restoration of human finger images under sparse-view imaging conditions. (A) Schematic diagram illustrating the configuration of the finger imaging experiment under dense-view and sparse-view conditions. (B) Ground-truth image, with an ROI indicated by a red box. The panel on the right provides a magnified view of this ROI. (C) Reconstructions using the FBP method with reduced transducer elements (*n* = 32, 64, 128, and 256). The 4 panels on the right display magnified details corresponding to each sparse-view case. (D to F) Images restored by FD-UNet, LV-GAN, and TT-PADM, respectively. For each method, the 4 panels on the right show magnified ROIs (top left, *n* = 32; top right, *n* = 64; bottom left, *n* = 128; bottom right, *n* = 256), corresponding to the same region marked by the red box in (B). (G) Quantitative evaluation of the results on the test dataset. (H) Comparison of intensity profiles along the horizontal and vertical axes at the center of the images for the 64-view case. Human hand image was created with BioRender.

For the sparse-view human finger experiments, a circular transducer array with 512 elements, identical to the one used in the previous mouse study, was used, as depicted in Fig. 5A. The number of transducer elements was progressively reduced from 512 to 256, 128, 64, and 32 elements, with 512-element images serving as the ground truth (Fig. 5B). Images reconstructed using FBP (500 × 500 pixels) exhibit streak artifacts and distortions, particularly in the internal details, as the number of transducers decreases (Fig. 5C). For restoration with 32 and 64 detectors, FD-UNet and LV-GAN fail to fully suppress artifacts or recover the boundaries of the fingers, whereas TT-PADM effectively restores the structural details and eliminates artifacts, as shown in Fig. 5D to F. In addition, TT-PADM demonstrates performance improvements over the original SGM (see Note S5). Quantitative evaluations further confirm the superiority of TT-PADM, as it achieves the highest values for PSNR and SSIM on the finger dataset (Fig. 5G). To substantiate the method’s effectiveness, Fig. 5H presents intensity profiles along the central horizontal and vertical axes of the images in the 64-view case. These profiles show that the reconstruction generated by TT-PADM closely approximates the ground truth, with all local deviations remaining below 0.01.

**Fig. 6.**
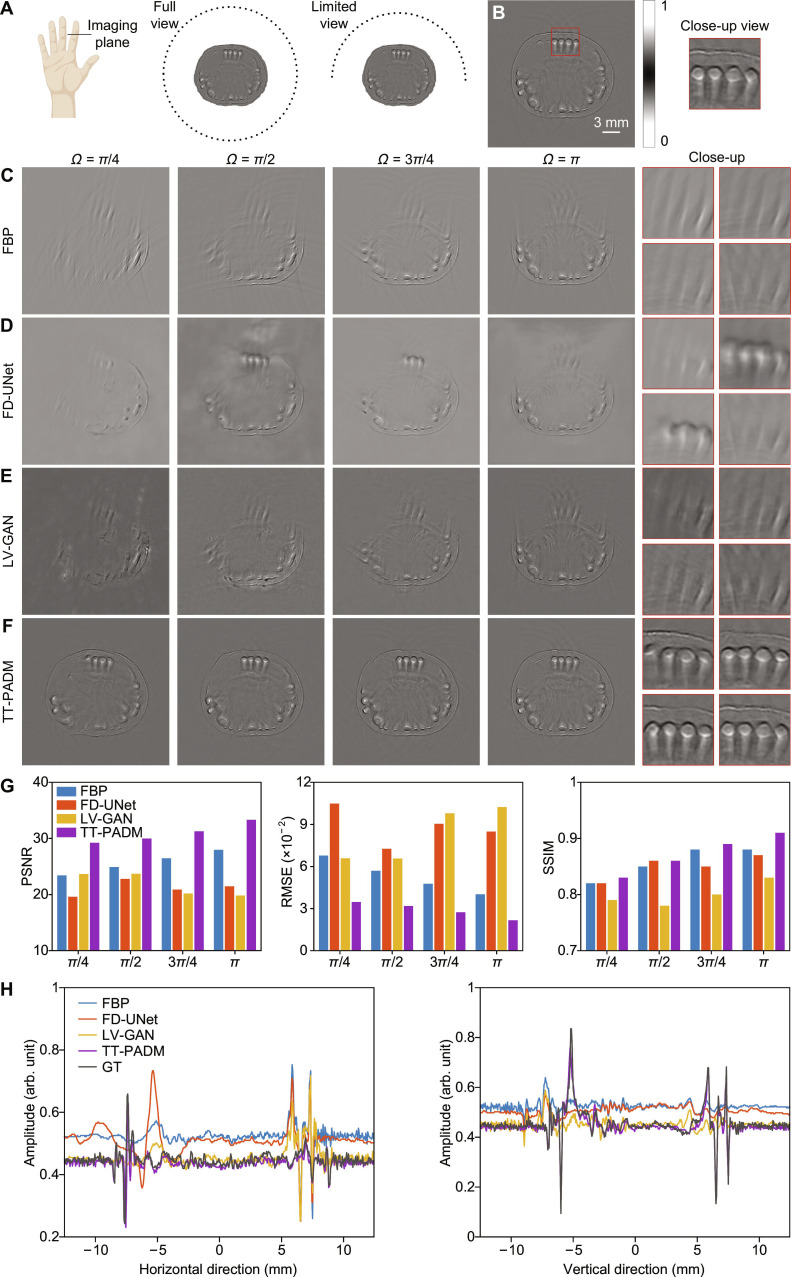
Restoration of human finger images under limited-view imaging conditions. (A) Schematic diagram illustrating the configuration of the finger imaging experiment under full-view and limited-view conditions. (B) Ground-truth image, with an ROI marked by a red box. The panel on the right provides a magnified view of this ROI. (C) Reconstructions using the FBP method with reduced angular coverage (*Ω* = *π*/4, *π*/2, 3*π*/4, and *π*). The 4 panels on the right display magnified details corresponding to each view angle. (D to F) Images restored by FD-UNet, LV-GAN, and TT-PADM, respectively. For each method, the 4 panels on the right display magnified ROIs (top left, *Ω* = *π*/4; top right, *Ω* = *π*/2; bottom left, *Ω* = 3*π*/4; bottom right, *Ω* = *π*), corresponding to the same region marked by the red box in (B). (G) Quantitative evaluation of the results on the test dataset. (H) Comparison of intensity profiles along the horizontal and vertical axes at the center of the images for the *π*/2-view case.

For the limited-view experimental studies, the transducer array geometry (Fig. 6A) follows the mouse imaging configuration, with angular coverage progressively reduced from full-view (2*π*) to *π*, 3*π*/4, *π*/2, and *π*/4. Corresponding full-view 512-element reconstructions serve as the ground truth (Fig. 6B). The limited-view images reconstructed using FBP (500 × 500 pixels) exhibit more pronounced loss of structural information compared to the in vivo mouse image reconstructions, primarily due to the complex acoustic heterogeneity introduced by the phalangeal structures (Fig. 6C). This anatomical complexity renders limited-view reconstruction considerably more challenging than sparse-view scenarios. Moreover, FD-UNet and LV-GAN fail to recover the boundaries of the fingers (Fig. 6D and E) and vessel details (highlighted by the red boxes in Fig. 6D and E) under all limited-view conditions. In contrast, TT-PADM mitigates distortion and markedly restores boundary and vein details, especially at view angles of *π*/4 and *π*/2, as highlighted by the red boxes in Fig. 6F. TT-PADM also shows performance gains over the original SGM (see Note S5). Consistent with the visual results, TT-PADM demonstrates superior performance in terms of PSNR, RMSE, and SSIM metrics (Fig. 6G) when compared to the other methods. At the *π*/2 limited-view angle, Fig. 6H presents intensity profiles along the central horizontal and vertical axes of the images. The profiles reconstructed by TT-PADM closely match the ground truth, demonstrating the method’s high restoration accuracy in highly heterogeneous media.

## Discussion

While limited-view and sparse-view transducer configurations reduce the cost of PAT systems, they often introduce severe image artifacts. Deep-learning-based postprocessing methods have been widely adopted to mitigate these issues due to their robust performance compared to conventional approaches. However, these methods require substantial representational capacity to compensate for considerable information loss during undersampled reconstructions. Although existing methods such as FD-UNet and LV-GAN yield promising results, their performance remains limited when applied to in vivo biological tissues, which introduce additional challenges such as low image contrast and acoustic heterogeneity. To address these limitations, we present TT-PADM, a diffusion-based framework that reconstructs high-quality images from highly undersampled PAT data by effectively modeling the underlying image distribution. Extensive evaluations on mouse embryo simulations, in vivo mouse studies, and human finger imaging demonstrate that TT-PADM consistently outperforms state-of-the-art approaches such as FD-UNet and LV-GAN in reconstruction accuracy and structural preservation, even under severe undersampling conditions.

This study also identifies several limitations of TT-PADM. First, the model’s inference speed remains a practical limitation, requiring approximately 37 s per image, compared with 0.54 s for FD-UNet and 0.37 s for LV-GAN, which may hinder its deployment in real-time imaging applications. This latency is primarily attributed to the iterative reverse diffusion process inherent to SGM. Second, the current model operates exclusively in the image domain, limiting its access to the richer, structurally preserved information available in the original signal domain. Third, the current validation is limited to circular transducer arrays and may not directly translate to linear arrays, which are commonly used in clinical practice. Future work will focus on several key directions: (a) exploring distilled sampling schedules [43], which leverage knowledge distillation to approximate multistep denoising with substantially fewer inference steps, together with lightweight module designs to accelerate inference; (b) extending the framework to a hybrid signal–image domain architecture, enabling multistage enhancement directly informed by raw measurement representations; (c) generalizing the approach to supporting a broader range of imaging scenarios involving complex tissue acoustic properties; and (d) adapting TT-PADM to clinical linear-array configurations through unsupervised or self-supervised learning strategies. The latter aims to address the limited availability of paired ground truth caused by alignment challenges between linear- and circular-array imaging planes, while improving robustness to directional artifacts and acoustic heterogeneity characteristic of linear-array systems.

In conclusion, we present TT-PADM, a novel image enhancement framework that integrates a time-driven transformer within an SGM to achieve high-quality PAT reconstruction from undersampled data. The core innovations of TT-PADM include the introduction of the TMTA mechanism and the GCFN module. The TMTA mechanism replaces standard self-attention with parameter-efficient channel attention and depth-wise convolution, reducing parameters by over 80% while preserving global context modeling (see Note S1 for analysis of parameter counts). Meanwhile, the GCFN module selectively refines features through a gating strategy. By balancing high reconstruction fidelity with computational efficiency, TT-PADM provides a robust and scalable solution for improving image quality in sparse-sampling PAT. In addition, it demonstrates strong performance under challenging in vivo imaging conditions, including low contrast and pronounced acoustic heterogeneities. These results highlight its capacity for generalization beyond idealized settings, positioning TT-PADM as a promising pathway toward clinical adoption and setting a new benchmark for generative model-based image restoration.

## Materials and Methods

The core of the proposed methodology for enhancing PAT images lies in the development of an efficient SGM integrated with time-driven transformer networks. This approach aims to retain the superior performance of transformers while simultaneously reducing computational overhead. The “Image reconstruction in PAT” section begins with an overview of the PAT forward problem and a conventional inverse image reconstruction technique. The “Photoacoustic diffusion model” section elaborates on the SGM, encompassing both the forward and reverse processes, as described by SDEs. The “Time-driven transformer network” section provides a detailed discussion of the time-driven transformer and its constituent components. Finally, the “Experimental setup and image dataset generation” and “TT-PADM training strategies” sections address the experimental setup used in this study and practical implementation aspects, including insights into the training and test datasets.

### Image reconstruction in PAT

Photoacoustic signals are generated on the basis of the photoacoustic effect, resulting from the irradiation of tissue with a nanosecond laser pulse. Accordingly, the photoacoustic pressure *p*(**r**, *t*) at position **r** and time *t* satisfies the wave equation [8]:

$$\nabla^{2}p\left(r,\;t\right)-\frac{1}{v^{2}}\frac{\partial^{2}}{\partial t^{2}}p\left(r,\;t\right)=-\frac{\beta }{C_{p}}\frac{\partial }{\partial t}H\left(r,\;t\right),$$ (1)

where *v* represents the speed of sound, *β* is the isobaric thermal volume expansion coefficient, *C_p_* is the specific heat capacity at constant pressure, and *H*(**r**, *t*) denotes the heat source. The FBP algorithm, proposed by Xu and Wang [10], is widely used for reconstructing the initial photoacoustic pressure due to its efficiency and robustness. For ideals detection geometries, FBP can be formulated using Green’s function as follows [8,10]:

$$p_{0}\left(r_{s}\right)=\int_{\Omega }b\left(r_{d},\;t\right)\delta \left(t-\frac{|r_{s}-r_{d}|}{v}\right)\frac{\delta \Omega }{\Omega },$$ (2)

where **r**_**s**_ and **r**_d_ represent the positions of the photoacoustic sources and detectors, respectively, *Ω* denotes the solid angle of the detection surface, *dΩ* is the solid angle subtended by an element, and the back-projection term *b*(**r**_d_*,t*) is given by the following:

$$b\left(r_{d},\;t\right)=2\left[p\left(r_{d},\;t\right)-t\frac{\partial p\left(r_{d},\;t\right)}{\partial t}\right].$$ (3)

A common method for calculating the time domain derivative in Eq. (3) involves multiplying the frequency domain data by *jω*, which allows ∂*p*(**r**_d_, *t*)/∂*t* to be approximated as a high-pass ramp filter in practice. However, directly applying this filter may introduce high-frequency ringing artifacts. To address this, a smoothing band-pass filter *W* is used during signal preprocessing, such as a third-order Butterworth filter with a bandwidth of 0.5 to 10 MHz. Thus, Eq. (3) can be rewritten as follows [8,10]:

$$b\left(r_{d},\;t\right)=2F^{-1}\left\{WF\left[p\left(r_{d},\;t\right)\right]-\text{tjωW}F\left[p\left(r_{d},\;t\right)\right]\right\},$$ (4)

where F and $F^{-1}$ represent Fourier transform and its inverse, respectively.

### Photoacoustic diffusion model

Diffusion models, such as SGM [31] and DDPM [32], conceptualize data generation as a Markov-chain-based denoising sequence. SGM generalizes this process within an SDE framework, establishing a continuous formalism for both noise injection and reversal. In contrast to DDPM, SGM provides a more rigorous theoretical foundation through its SDE formulation, which facilitates adaptable noise scheduling, accelerated sampling, and improved robustness in solving ill-posed inverse problems.

**Fig. 7.**
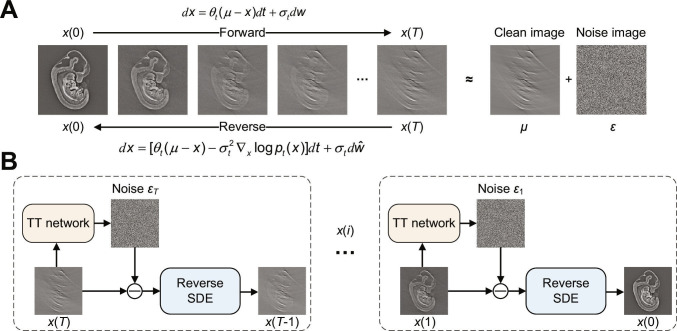
Principle of the TT-PADM image restoration method. (A) Training phase: The forward SDE models the degradation process from a high-quality image *x*(0) to its degraded counterpart *μ*, by diffusing *x*(0) toward a noisy version *μ + ε*. The reverse SDE process is then simulated to restore high-quality images. (B) Restoration phase: Time-driven transformers are used to approximate the noise, facilitating the simulation of the reverse SDE process. TT, time-driven transformer.

Within this framework, SGM progressively transforms an image distribution into a tractable prior via systematic noise introduction, while the corresponding reverse process reconstructs the original distribution through iterative denoising [31]. To transform a sparse-view or limited-view image into a high-quality image, the forward SDE function is defined as follows [44]:

$$\mathrm{dx}=\theta_{t}\left(\mu -x\right)\mathrm{dt}+\sigma_{t}\mathrm{dw},$$ (5)

where *x* represents a stochastic process, *μ* is the state mean corresponding to a sparse-view or limited-view photoacoustic image, *θ_t_* and *σ_t_* denote preset time-varying positive parameters defining the rate of mean reversion and the level of stochastic volatility, respectively, and *w* is a standard Wiener process. According to the SDE proposed in [44], the time-varying parameters satisfy $\sigma_{t}^{2}/\theta_{t}=2\lambda^{2}$ at all times *t*, where *λ* represents the noise level. Therefore, given the initial state *x*(0), the solution to the SDE is as follows:

$$x\left(t\right)=\mu +\left[x\left(0\right)-\mu \right]e^{-{\bar{\theta }}_{0:t}}+\int_{0}^{t}\sigma_{z}e^{-{\bar{\theta }}_{z:t}}\mathrm{dw}\left(z\right),$$ (6)

where ${\bar{\theta }}_{0:t}≔\int_{0}^{t}\theta_{z}\mathrm{dz}.$ In addition, the marginal distribution of state *x*(*t*), denoted as, *p_t_*(*x*), can be computed as follows [31]:

$$\begin{array}{l} \;p_{t}\left(x\right)=N\left(x\left(t\right)|m_{t},\;v_{t}\right),\\ \;m_{t}≔\mu +\left(x\left(0\right)-\mu \right)e^{-{\bar{\theta }}_{0:t}},\\ \;v_{t}≔\lambda^{2}\left(1-e^{-2{\bar{\theta }}_{0:t}}\right), \end{array}$$ (7)

where 𝒩 represents a normal distribution and *m_t_* and *v_t_* represent the time-varying mean and variance, respectively. As time *t* progresses, *m_t_* and *v_t_* gradually converge into *μ* and *λ*^2^, respectively. In practice, *μ* and *x*(0) are set to the pair of a sparse-view or limited-view image and its full-view counterpart, as depicted in Fig. 7A. A high-quality image can then be recovered through the time-reversed SDE, which is given by the following [31]:

$$\mathrm{dx}=\left[\theta_{t}\left(\mu -x\right)-\sigma_{t}^{2}\;\nabla_{x}\text{log}\;p_{t}\left(x\right)\right]\mathrm{dt}+\sigma_{t}d\hat{w},$$ (8)

where $\hat{w}$ represents a time-reversed Wiener process and $\nabla_{x}\text{log}\;p_{t}\left(x\right)$ is the score function. According to Eq. (7), the score function $\nabla_{x}\text{log}p_{t}\left(x\right)$ can be expressed as follows:

$$\nabla_{x}\text{log}\;p_{t}\left(x\right)=-\frac{x\left(t\right)-m_{t}}{v_{t}}.$$ (9)

Following the formulation in [44], we reparameterize $x\left(t\right)=m_{t}+\sqrt{v_{t}}\varepsilon_{t}$, where $\varepsilon_{t}$ denotes the standard Gaussian noise 𝒩(0, *I*) and *I* is the identity matrix. As a result, the score function can be rewritten as

$$\nabla_{x}\text{log}\;p_{t}\left(x\right)=-\frac{\varepsilon_{t}}{\sqrt{v_{t}}}.$$ (10)

Finally, a time-dependent transformer network *s_ϕ_*(*x*(*t*), *μ*, *t*) is used to approximate the score function at each time step. In practice, this is achieved by approximating the noise using a noise network, as shown in Fig. 7B, defined by

$$L\left(\phi \right)≔\underset{\phi }{\text{arg}\text{min}}\sum_{i=1}^{T}∥s_{\phi }\left(x\left(i\right),\;\mu ,\;i\right)-\varepsilon_{i}{∥}_{2}^{2},$$ (11)

where *L* denotes the mean square error loss function and *ϕ* represents the parameters of the network. Once trained, the network *s_ϕ_* can be used to reconstruct high-quality images by sampling a noise state and iteratively resolving the time-reversed SDE.

### Time-driven transformer network

The performance of the SGM heavily relies on the capacity of the time-dependent neural network to accurately estimate the score function. A commonly used neural network for noise prediction is the convolutional U-Net [45], which incorporates residual blocks [46] and attention mechanisms such as spatial [47] and channel attention [48]. However, because of a limited receptive field, convolutional layers constrain the capacity to model long-range pixel correlations. Recently, vision transformer blocks, such as diffusion transformer (DiT) [49] and vision transformer (ViT) [50], have been integrated into diffusion models, leading to substantial performance improvements. However, the computational cost associated with pure transformer blocks escalates quadratically with the number of image patches, making them less practical for processing high-resolution images.

**Fig. 8.**
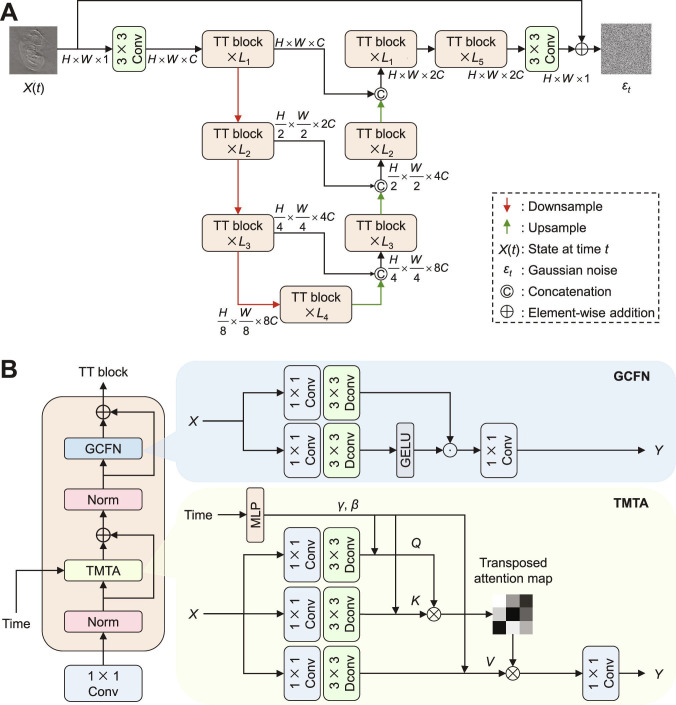
Architecture design of the time-driven transformer network. (A) Overall structure of time-driven transformer network. *X*(*t*) represents the features in state *t*. The network consists of 5 layers, with each layer containing several time-driven transformer blocks. (B) Internal architecture of the time-driven transformer block. The time-driven transformer block includes 2 core modules: the TMTA and the GCFN. TMTA uses channel attention instead of spatial attention to compute the query (*Q*), key (*K*), and value (*V*) through 1 × 1 convolution (Conv) and 3 × 3 depth-wise convolution (Dconv) layers, while integrating time conditions via a multilayer perceptron (MLP) in the computation of multihead attention. GCFN enables controlled feature transformation through the GELU.

To preserve the efficacy of transformer blocks while mitigating computational costs, we introduce a time-driven transformer for noise prediction, inspired by the Restormer architecture [51]. This network uses an encoder–decoder structure with skip connections to facilitate multi-scale feature aggregation, as illustrated in Fig. 8A. The network consists of 5 hierarchical layers, each containing multiple time-driven transformer blocks that progressively process features at different spatial resolutions. In each block, 2 key components, TMTA and GCFN, are introduced to replace the multihead self-attention and feedforward network (FN) layers in the standard transformer [49,50].

#### Time-driven multihead transposed attention

TMTA utilizes a channel-wise attention mechanism to establish global dependencies across all pixels in the input feature maps, effectively capturing long-range spatial interactions through the computation of transposed attention weights. Compared to a standard multihead self-attention layer [49,50], TMTA introduces 3 key modifications, as shown in Fig. 8B. First, time is incorporated in the computation of query (*Q*), key (*K*), and value (*V*) arrays via a multilayer perception to transform the time *t* into channel-wise scale *γ* and shift arrays *β* as priors. Second, the combination of 1 × 1 convolution and depth-wise convolutions is introduced to generate *Q*, *K*, and *V*, reducing the number of parameters as described by the following:

$$\begin{array}{l} Q=W_{d}^{Q}\left[W_{c}^{Q}X\left(\gamma_{Q}+1\right)+\beta_{Q}\right],\\ K=W_{d}^{K}\left[W_{c}^{K}X\left(\gamma_{K}+1\right)+\beta_{K}\right],\\ V=W_{d}^{V}\left[W_{c}^{V}X\left(\gamma_{V}+1\right)+\beta_{V}\right], \end{array}$$ (12)

where $X\in {\mathbb{R}}^{H\times W\times C}$ is the input feature, *H*×*W* represents the spatial dimension, and *C* denotes the number of channels. $W_{d}^{\left(\cdot \right)}$ denotes 3 × 3 depth-wise convolution, and $W_{c}^{\left(\cdot \right)}$ represents 1 × 1 convolution. Third, attention features are computed across channels, rather than spatial dimensions, and separate attention maps are trained for each channel. Therefore, TMTA can be defined as

$$\begin{array}{c} \begin{array}{c} Y=W_{p}\text{Attention}\left(\bar{Q},\bar{K},\bar{V}\right),\\ \text{Attention}\left(\bar{Q},\bar{K},\bar{V}\right)=\bar{V}\cdot \text{softmax}\left(\bar{K}\cdot \bar{Q}/\alpha \right), \end{array} \end{array}$$ (13)

where $Y\in {\mathbb{R}}^{H\times W\times C}$ is the output feature; $\bar{Q}\in {\mathbb{R}}^{\mathrm{HW}\times C}$, $\bar{K}\in {\mathbb{R}}^{C\times \mathrm{HW}}$, and $\bar{V}\in {\mathbb{R}}^{\mathrm{HW}\times C}$ are the reshaped matrices from the original input images, respectively; and *α* is a learnable scaling parameter to constrain the magnitude of the dot product before applying the softmax function.

Through these 3 architectural innovations, TMTA effectively captures global pixel-wise dependencies in input features and seamlessly integrates temporal conditioning into attention computation. The module achieves a substantial parameter reduction of 80% per attention block compared to standard transformer architectures, primarily attributable to the strategic adoption of depth-wise convolutions and channel-oriented attention mechanisms (see Note S1 for analysis of parameter counts). This design preserves modeling capacity while significantly alleviating computational overhead, making it particularly suitable for high-resolution image reconstruction tasks within the diffusion model framework.

#### Gated convolutional feedforward network

GCFN enables selective feature refinement through a gating mechanism that selectively attenuates less informative components while enhancing the propagation of task-relevant features across network layers. A common practice on FN is to process each pixel separately using convolutions and applying a nonlinear activation function to increase model complexity. In contrast, GCFN uses a gating mechanism, such as the Gaussian error linear unit (GELU), and depth-wise convolutions to replace the nonlinear activation and convolutions, as shown in Fig. 8B. The formulation for GCFN is given by the following:

$$\begin{array}{c} Y=W_{c}^{3}\text{Gating}\left(X\right),\\ \text{Gating}\left(X\right)=\text{GELU}\left(W_{d}^{1}W_{c}^{1}X\right)⊙W_{d}^{2}W_{c}^{2}X, \end{array}$$ (14)

where ⊙ stands for element-wise multiplication. GCFN provides an efficient and expressive alternative to standard FNs, enabling selective feature transformation through gated mechanisms while ensuring parameter efficiency via depth-wise convolution.

### Experimental setup and image dataset generation

The photoacoustic images used for training and testing were divided into experimental and simulation datasets to evaluate the performance of the proposed method. In the experimental setup, a pulsed laser system with a wavelength of 750 nm and a repetition rate of 10 Hz was used to generate photoacoustic signals. A ring-shaped ultrasound transducer with a radius of 40 mm, a center frequency of 5 MHz, and a bandwidth of 70% was utilized. The system included a data acquisition setup operating at a sampling frequency of 62.5 MHz for receiving and processing the photoacoustic signals (see Fig. S1). The animal experiments were conducted in accordance with the National Institutes of Health Guide for the Care and Use of Laboratory Animals, with approval from the Institutional Animal Care and Use Committee of the University of Science and Technology of China (protocol number USTCACUC1803065). The study involving human participants was approved by the Institutional Review Board of the First Affiliated Hospital of the University of Science and Technology of China (IRB no. 2022-ky357) and conducted in accordance with the principles of the Declaration of Helsinki. For the simulation dataset, the k-Wave toolbox, developed by Treeby and Cox [52], was used to simulate photoacoustic signal generation, propagation, and acquisition. The simulation parameters for the transducer array were matched to those used in the experimental setup.

A partitioning strategy was used in which 80% of the dataset was allocated for model training, allowing the model to learn intricate internal features and patterns. Ten percent of the images were reserved for model validation to ensure effective generalization and performance optimization. The remaining 10% of the images were used for model testing, enabling a robust evaluation of the model’s predictive capabilities.

### TT-PADM training strategies

For all tasks, the following settings are applied: a batch size of 2, an Adam optimizer with *β*_1_ = 0.9 and *β*_2_ = 0.99, an initial learning rate of 3 × 10^−5^ with cosine-scheduler decay, a noise level set to 50, and a fixed number of states set to 200. All deep learning models are implemented using PyTorch (version 2.0.1) on a Microsoft Windows 10 operating system. The training was conducted on a workstation equipped with an Intel Xeon Gold 6226R CPU at 3.5 GHz and a single NVIDIA TITAN GPU with 24 GB of memory. After training, TT-PADM typically takes 37 s to process a single PAT image with a resolution of 500 × 500 pixels in this study.

## Data Availability

All data generated or analyzed during this study are included in this article. Further requests can be made to the corresponding author.

## Supplementary Materials

Supplementary 1 Figs. S1 to S5 Notes S1 to S5
