# Supplementary material for: TT-PADM: A Time-Driven Transformer Diffusion Model for Robust Sparse-View and Limited-View Photoacoustic Tomography
Source: BME Front. 2026 Mar 2;7:0237. doi: 10.34133/bmef.0237 (PMC12951294; doi:10.34133/bmef.0237)
Supplement: Supplementary 1 — Figs. S1 to S5 Notes S1 to S5 [file bmef.0237.f1.docx]

**Front Matter**

Title

Supplementary materials for TT-PADM: a time-driven transformer diffusion model for robust sparse-view and limited-view photoacoustic tomography

**Authors**

Jiawei Zheng^1,2,3,†^, Wende Dong^4,†^, Junjun Sun^3,5,†^, Qingfei Song^6^, Xiaohua Jiang^7^, Sheng Wang^1^, Songde Liu^1,6,*^, and Chao Tian^1,3,6,8,*^

**Affiliations**

^1^Department of Anesthesiology, the First Affiliated Hospital of USTC, Division of Life Sciences and Medicine, University of Science and Technology of China, Hefei, Anhui 230001, China

^2^Institute of Advanced Technology, University of Science and Technology of China, Hefei, Anhui 230026, China

^3^Institute of Artificial Intelligence, Hefei Comprehensive National Science Center, Hefei, Anhui 230088, China

^4^College of Automation Engineering, Nanjing University of Aeronautics and Astronautics, Nanjing, Jiangsu 211106, China

^5^School of Artificial Intelligence, Anhui University, Hefei, Anhui 230601, China

^6^School of Engineering Science, University of Science and Technology of China, Hefei, Anhui 230026, China

^7^Department of Obstetrics and Gynecology, The First Affiliated Hospital of USTC, Division of Life Sciences and Medicine, University of Science and Technology of China, Hefei, Anhui, 230001, China

^8^Anhui Province Key Laboratory of Biomedical Imaging and Intelligent Processing, Institute of Artificial Intelligence, Hefei Comprehensive National Science Center, Hefei, Anhui 230088, China

^*^Address correspondence to: [liusde@ustc.edu.cn](mailto:liusde@ustc.edu.cn) (S.L.) and [ctian@ustc.edu.cn](mailto:ctian@ustc.edu.cn) (C.T.)

^†^These authors contributed equally to this work.

**The PDF file includes:**

**Fig. S1**. Schematic diagram of the custom photoacoustic tomography (PAT) imaging system.

**Note S1**: Comparison of parameter counts in the time-driven transformer used in this study and conventional transformers

**Note S2**: Evaluation metrics

**Note S3**: Comparative study of the proposed TT-PADM and SGM on mouse embryo simulations

**Note S4**: Comparative study of the proposed TT-PADM and SGM on in vivo mouse experiments

**Note S5**: Comparative study of the proposed TT-PADM and SGM on human finger experiments

**Additional references**


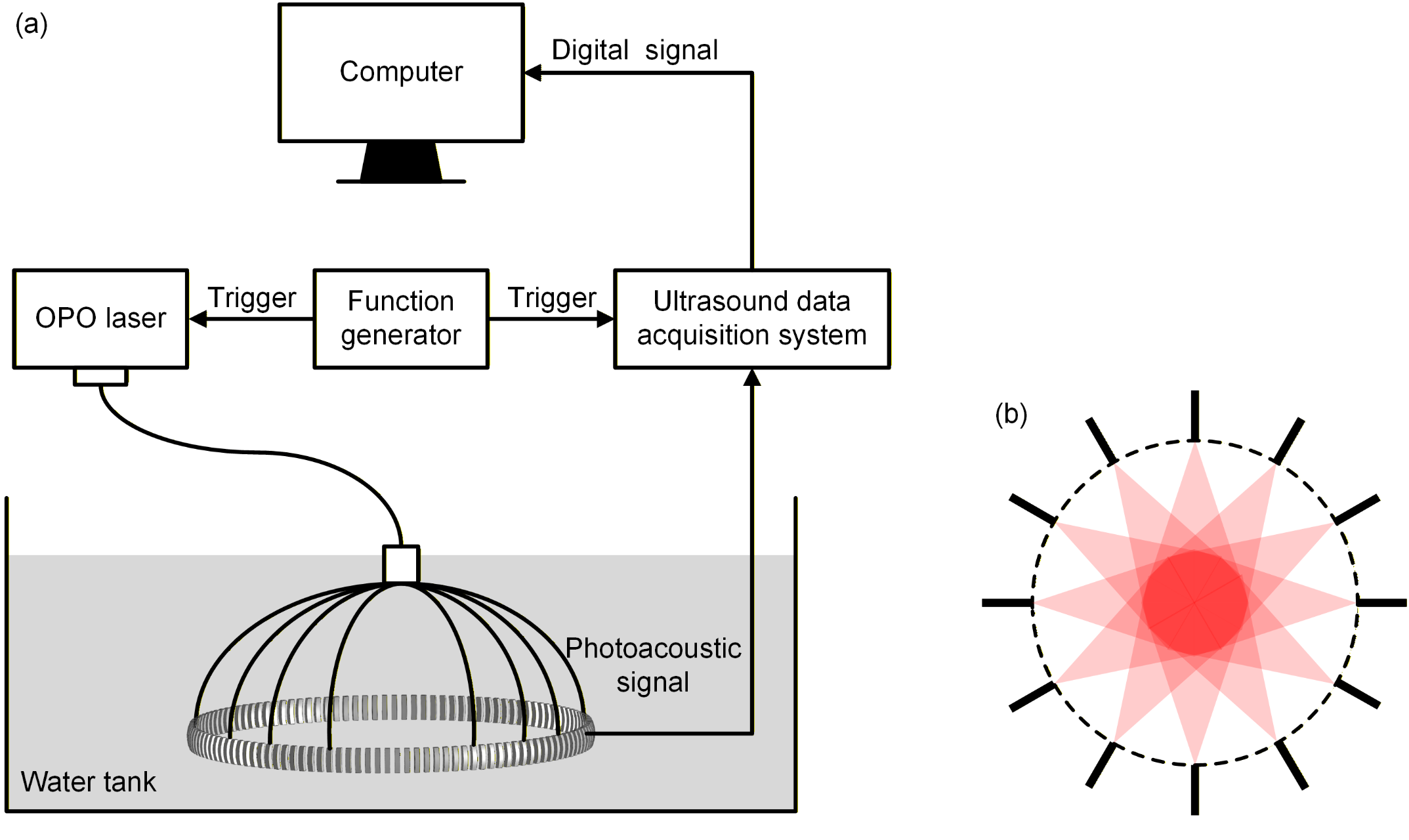


**Fig. S1** Schematic diagram of the custom photoacoustic tomography (PAT) system. (a) Schematic overview of the system. (b) Schematic diagram of the optical fiber-based illumination system.

**Note S1: Comparison of parameter counts in the time-driven transformer used in this study and conventional transformers**

In standard transformer architectures for image processing, the primary components include multi-head self-attention (MSA) layers and feed-forward networks (FNs), which are typically implemented as multilayer perceptrons (MLPs)[1, 2]. The computational complexity in transformer blocks is primarily driven by the MSA layers, which capture long-range dependencies through a significant number of 3×3 convolutional layers [see Fig. S2(a)]. To maintain the efficacy of transformer blocks while reducing computational costs, the proposed time-driven transformer (TT) integrates multi-head transposed attention (TMTA) and a gated convolutional feed-forward network (GCFN), which reduces parameter counts mainly by replacing standard 3×3 convolutions with 3×3 depth-wise convolutions [see Fig. S2(b)].

Since the MSA layers account for the majority of computational complexity, we focus on comparing MSA and TMTA. In conventional transformers, MSA uses three separate 3×3 convolutions to generate the query (*Q*), key (*K*), and value (*V*) features, along with one 1×1 convolution to process the output. The parameter count for each convolution layer is *n*×*n*×*C*^2^, where *n*×*n* is the kernel size and *C* is the number of feature channels. Thus, the total parameter count for MSA is 28×*C*^2^. In contrast, TMTA in the time-driven transformer uses a combination of 1×1 convolutions and 3×3 depth-wise convolutions to compute *Q*, *K*, and *V*. The depth-wise convolution requires 3×3×*C* parameters per kernel. Excluding the time processing components, the total parameter count for TMTA is 27×*C*+3×*C*^2^. Given that the number of initial channels typically ranges from 16 to 64 and increases with network depth, TMTA achieves a parameter reduction of at least 80% compared to standard MSA.


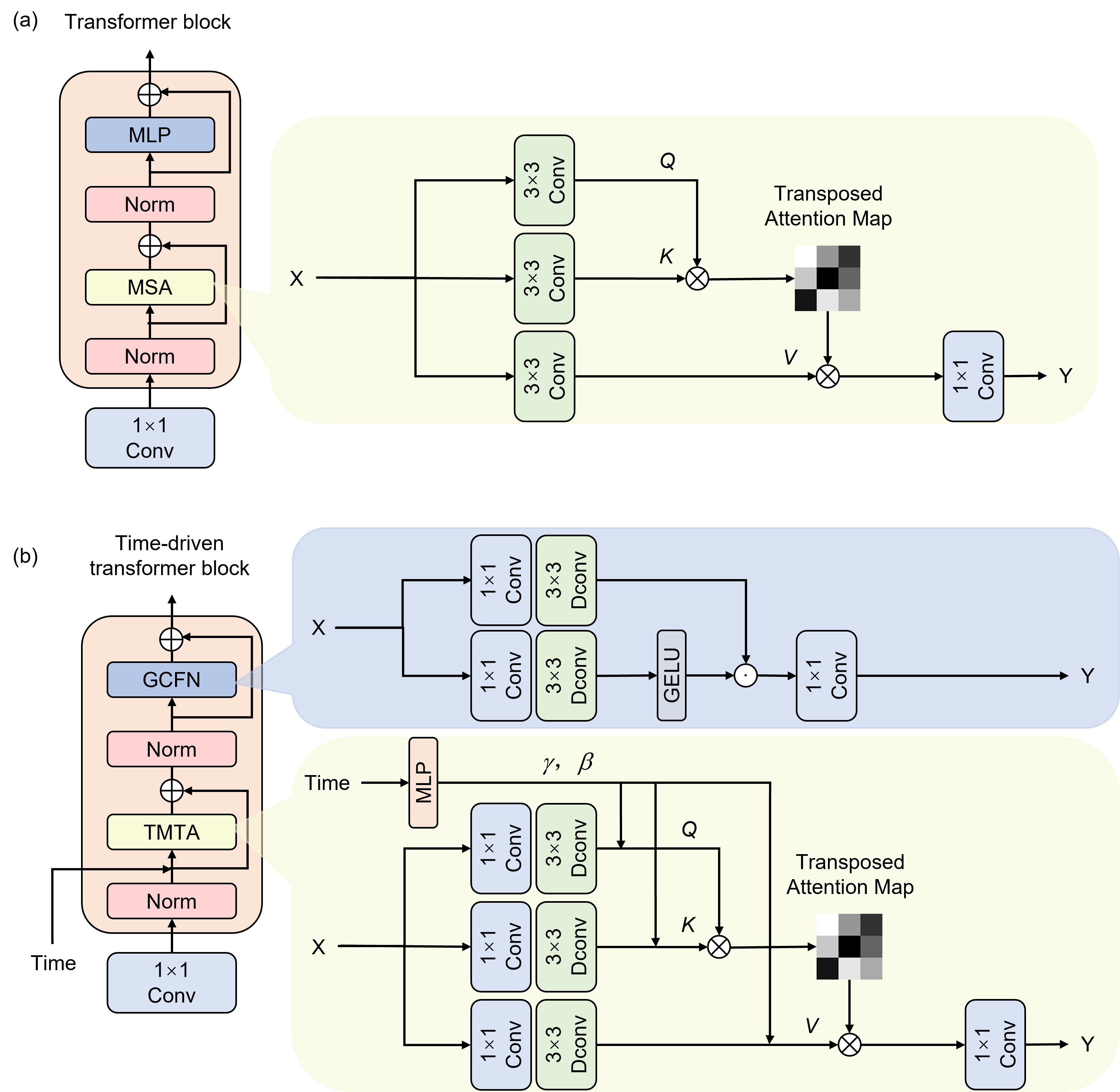


**Fig. S2** Architectures of a conventional transformer block (a) and the time-driven transformer (TT) block employed in this study (b).

**Note S2: Evaluation metrics**

To quantitatively assess the performance of different reconstruction methods, we employed three widely-used metrics: Peak Signal-to-Noise Ratio (PSNR), Root Mean Square Error (RMSE) and Structural Similarity Index (SSIM) [3]. The PSNR is defined as:

$\begin{matrix} \text{PSNR}=10\log_{10}\frac{\left[\max(x)\right]^{2}}{\text{MSE(x,y)}}, \\ \text{MSE}=\frac{1}{mn}\sum_{i=0}^{m-1} \sum_{j=0}^{n-1} [y(i,j)-x(i,j)]^{2}, \end{matrix}$ ()

where *x* and *y* denote the image and the reference image, respectively; max(*x*) stands for the maximum value of the image *x*; MSE is the mean square error; and *m* and *n* are the image dimensions. The RMSE is defined as:

$\text{RMSE}=\sqrt{\text{MSE}(x,y)}.$ ()

The SSIM is defined as:

$\text{SSIM}=\frac{(2\mu_{y}\mu_{x}+C_{1})(2\sigma_{xy}+C_{2})}{(\mu_{y}^{2}+\mu_{x}^{2}+C_{1})(\sigma_{y}^{2}+\sigma_{x}^{2}+C_{2})},$ ()

where *μ_y_* and *μ_x_* are the means of the reference image *y* and image *x*, respectively; *σ_x_* and *σ_y_* represent the variances of the two images; *σ_xy_* denotes the covariance between the two images; $C_{1}=[0.01\max(y)]^{2}$ and $C_{2}=[0.03\max(y)]^{2}$ are two positive constants used to stabilize the division with weak denominator values.

**Note S3: Comparative study of the proposed TT-PADM and SGM on mouse embryo simulations**

To further validate the advantages of integrating a time-driven transformer into the score-based generative model (SGM), we compared the performance of the Time-driven Transformer-based Photoacoustic Diffusion Model (TT-PADM) with the original SGM, using a U-Net as the noise prediction network. Both methods employ stochastic differential equations (SDEs) to establish a bidirectional mapping between spatially undersampled and full-view images. However, the convolutional layers in U-Net are restricted to local receptive fields, limiting their ability to capture long-range dependencies. This constraint hampers the network's capacity to accurately predict noise across various states, which may result in suboptimal performance when enhancing severely sparse-view and limited-view images, both in simulation and experimental settings.

To validate this hypothesis, we conducted a comparative evaluation using both sparse-view and limited-view mouse embryo simulations. The simulation setup was consistent with that described in the main manuscript [see Fig. S3(a)–(b)]. For sparse-view reconstruction, the number of transducer elements was reduced from 512 to 64 and 32; for the limited-view case, the angular range was decreased from 2π to π/2 and π/4. As shown in Fig. S3(c)–(d), although the original SGM accurately recovers embryo boundaries under both conditions, it fails to preserve fine internal structural details under the 32-element and π/4-view configurations. In contrast, TT-PADM demonstrates significant improvements in reconstructing vascular structures and boundary details across all settings. Furthermore, as summarized in Fig. S3(e), TT-PADM consistently outperforms the original SGM in terms of PSNR, RMSE, and SSIM, confirming that replacing the U-Net with a time-driven transformer enhances the reconstruction quality for both sparse-view and limited-view imaging.


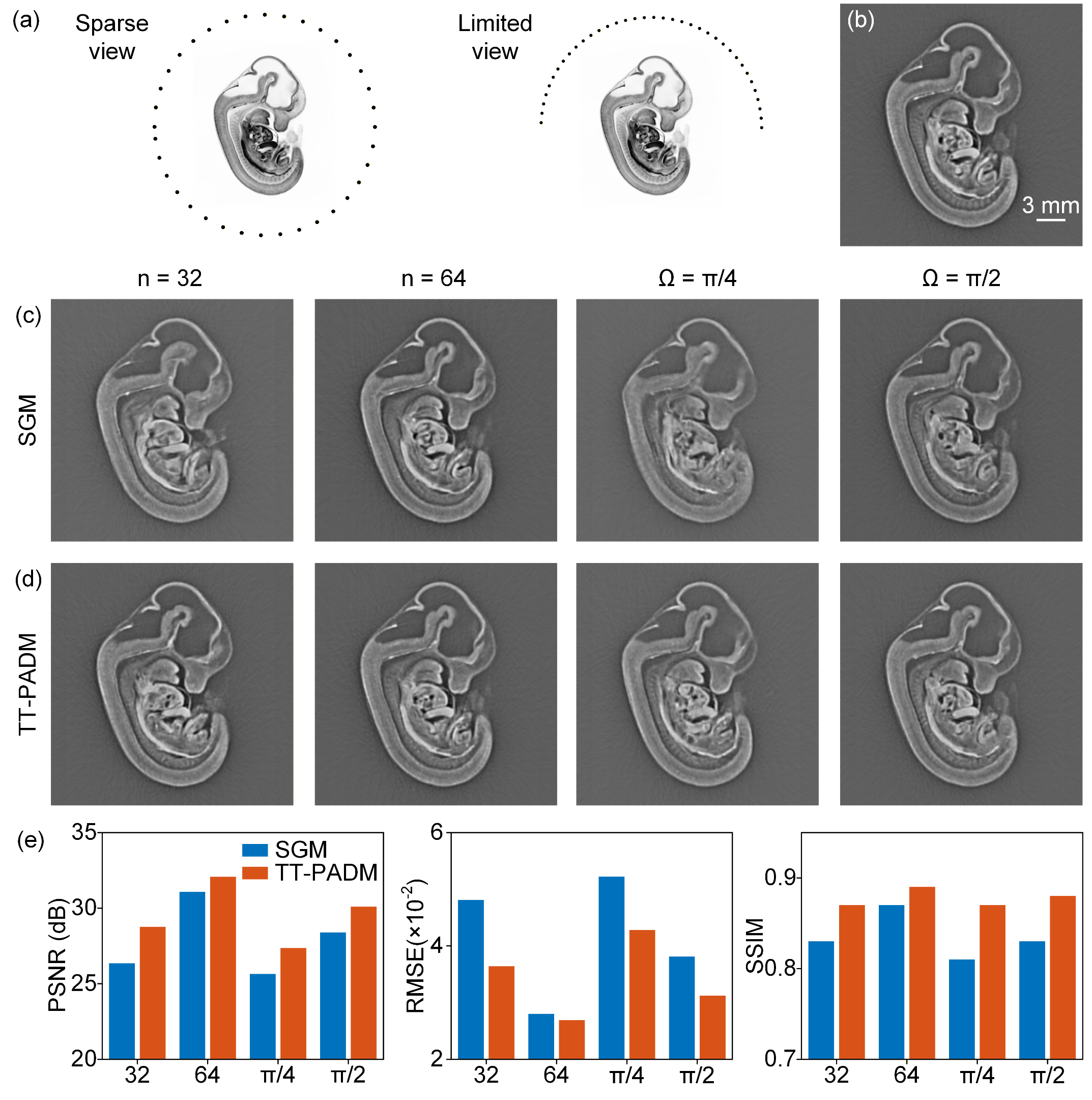


**Fig. S3** Restoration of mouse embryo images using SGM and TT-PADM under sparse-view and limited-view imaging conditions. (a) Schematic diagram illustrating the configuration of the mouse embryo imaging simulation under sparse-view and limited-view conditions. (b) Ground truth image. (c)-(d) Enhanced results from SGM and TT-PADM, respectively. (e) Quantitative evaluation of the results on the test dataset.

**Note S4: Comparative study of the proposed TT-PADM and SGM on in vivo mouse experiments**

To assess the performance of both methods in practical experiments, we conducted a comparative study using *in vivo* mouse experiments under sparse-view and limited-view conditions. The experimental setup [Fig. S4(a)–(b)] was consistent with the configuration described earlier in the main manuscript. Compared to the simulated embryo images, the *in vivo* mouse images exhibit lower contrast, making the enhancement task more challenging. In the sparse-view configuration, the number of transducer elements was reduced from 512 to 64 and 32, while in the limited-view case, the angular range was reduced from 2π to π/2 and π/4. As shown in Fig. S4(c)–(d), both methods perform well in recovering boundary and internal structures under sparse-view conditions. However, under the severely limited π/4-view condition, SGM fails to adequately recover the mouse boundary, which can be attributed to insufficient spatial sampling and low image contrast. In contrast, TT-PADM maintains robust performance across all acquisition conditions. This superiority is further validated by its consistently higher scores in PSNR, RMSE, and SSIM [Fig. S4(e)], confirming the advantage of integrating the time-driven transformer into the reconstruction framework.


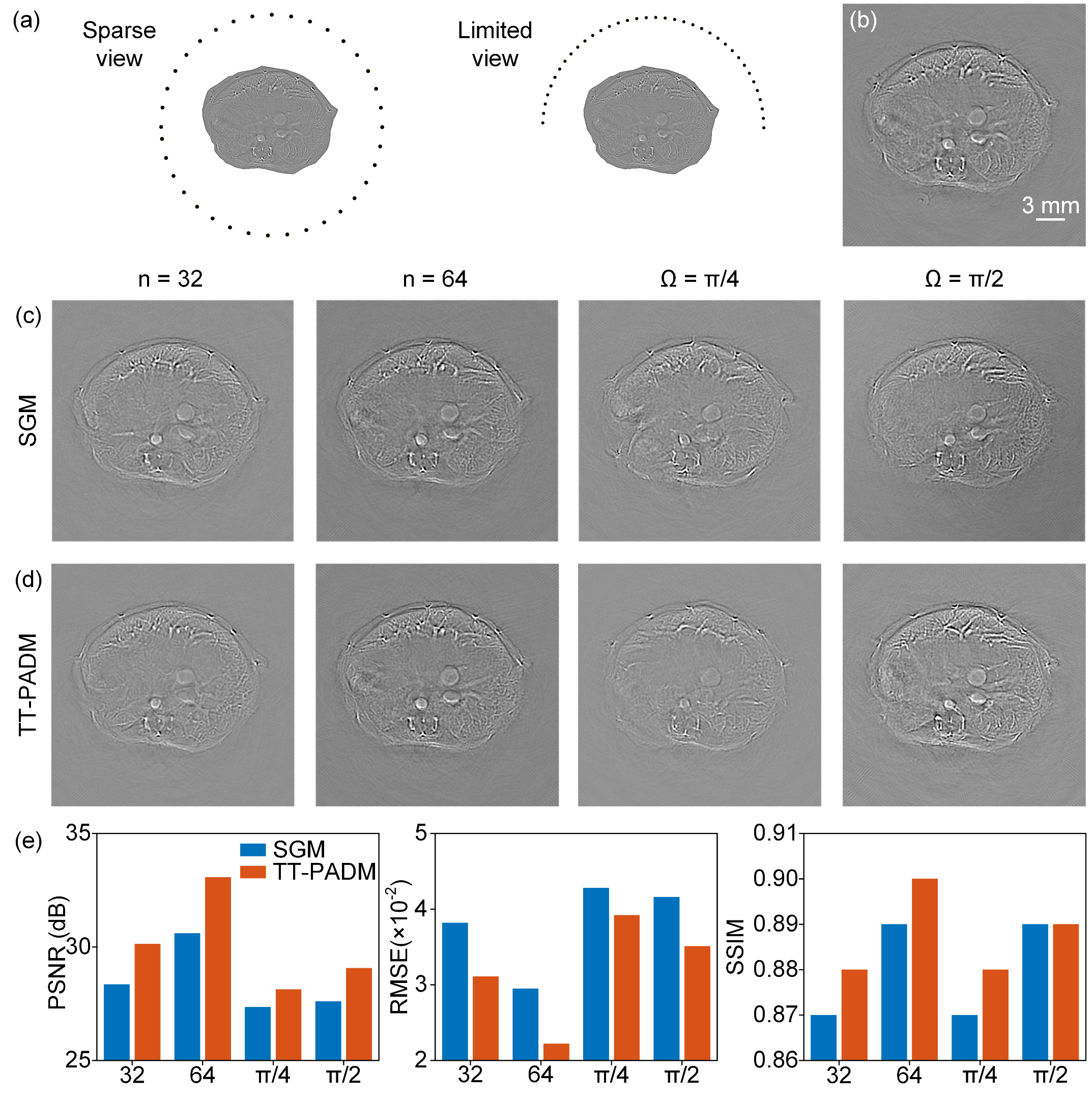


**Fig. S4** Restoration of *in vivo* mouse images using SGM and TT-PADM under sparse-view and limited-view imaging conditions. (a) Schematic diagram illustrating the configuration of the *in vivo* mouse imaging simulation under sparse-view and limited-view conditions. (b) Ground truth image. (c)–(d) Enhanced results from SGM and TT-PADM, respectively. (e) Quantitative evaluation of the results on the test dataset.

**Note S5: Comparative study of the proposed TT-PADM and SGM on human finger experiments**

In human finger experiments, spatially undersampled images exhibit greater structural information loss compared to *in vivo* mouse studies, due to the presence of highly heterogeneous media, such as phalanges, particularly under limited-view conditions. To evaluate whether the two methods can effectively handle such heterogeneous media in sparse-view and limited-view scenarios, we applied both approaches under the same experimental configuration [Fig. S5(a)–(b)] as described in the main manuscript. For sparse-view imaging, the number of transducer elements was reduced from 512 to 64 and 32, while for limited-view imaging, the angular range was restricted from 2π to π/2 and π/4. As shown in Fig. S5(c)–(d), both methods effectively suppress streak artifacts in the background and within the finger region. However, under severely limited sampling conditions (e.g., π/2 and π/4 views), SGM fails to accurately reconstruct the finger contour and detailed vessel structures. In contrast, TT-PADM consistently demonstrates robust performance in boundary recovery and artifact suppression across all acquisition settings. This advantage is further supported by its superior scores in PSNR, RMSE, and SSIM [Fig. S5(e)], highlighting the effectiveness of integrating the time-driven transformer into the reconstruction model.


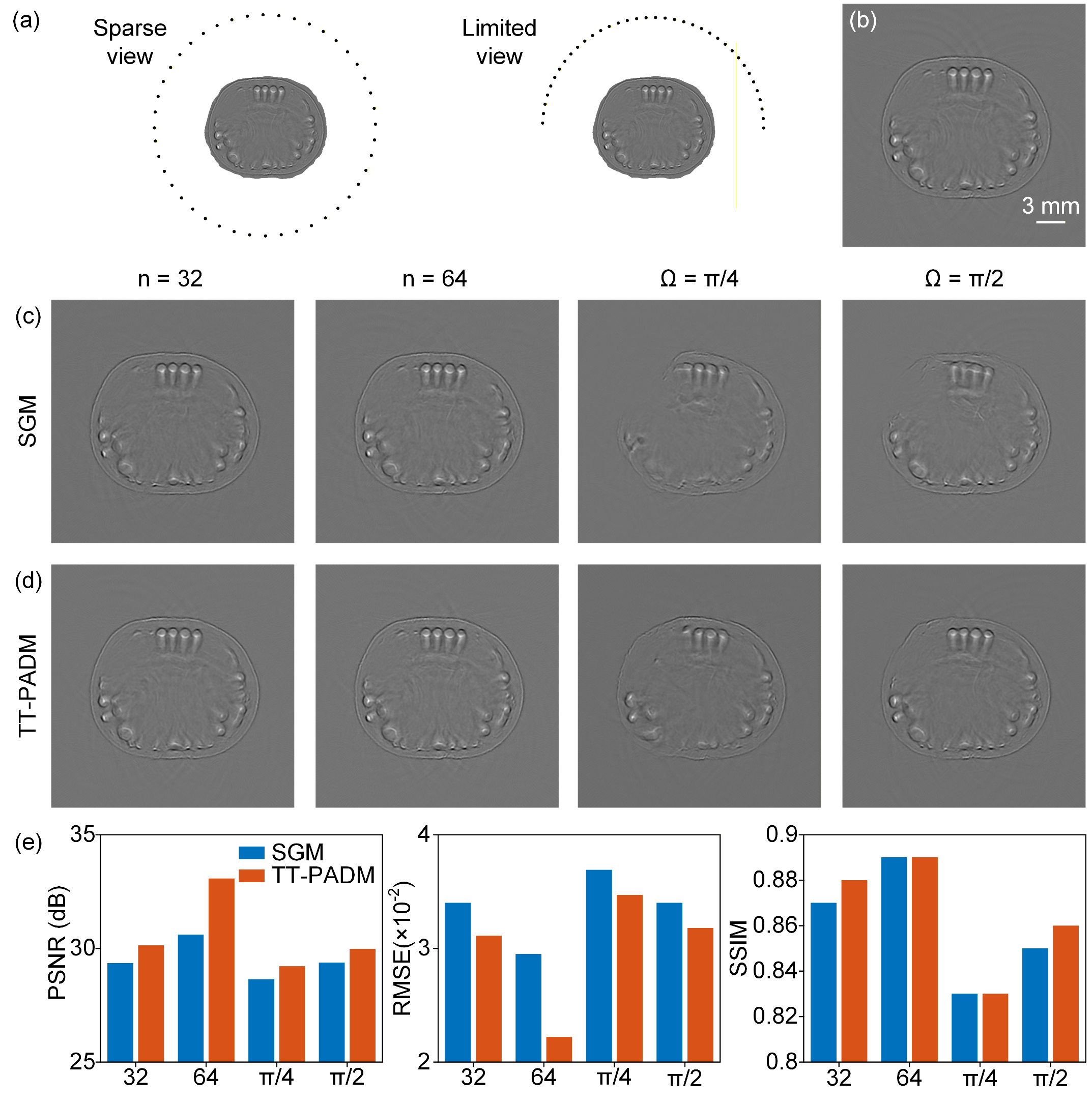


**Fig. S5** Restoration of human finger images using SGM and TT-PADM under sparse-view and limited-view imaging conditions. (a) Schematic diagram illustrating the configuration of the human finger imaging simulation under sparse-view and limited-view conditions. (b) Ground truth image. (c)–(d) Enhanced results from SGM and TT-PADM, respectively. (e) Quantitative evaluation of the results on the test dataset.

Additional references

[1] Bao F, Nie S, Xue K, Cao Y, Li C, Su H, et al., editors. All are worth words: A vit backbone for diffusion models. Proceedings of the IEEE/CVF Conference on Computer Vision and Pattern Recognition; 2023.

[2] Peebles W, Xie S, editors. Scalable diffusion models with transformers. Proceedings of the IEEE/CVF International Conference on Computer Vision; 2023.

[3] Wang Z, Bovik AC, Sheikh HR, Simoncelli EP. Image quality assessment: from error visibility to structural similarity. IEEE Transactions on Image Processing. 2004;13(4).
